# DroNER: Dataset for drone named entity recognition

**DOI:** 10.1016/j.dib.2023.109179

**Published:** 2023-04-25

**Authors:** Swardiantara Silalahi, Tohari Ahmad, Hudan Studiawan

**Affiliations:** Department of Informatics, Institut Teknologi Sepuluh Nopember, Surabaya, Indonesia

**Keywords:** NER dataset, Drone forensics, Drone dataset, Drone entity recognition, Digital forensics, Infrastructure

## Abstract

The dataset is constructed from the drone flight log messages extracted from publicly available drone image datasets provided by VTO Labs under the Drone Forensic Program. The entire process of building this dataset includes extraction, decryption, parsing, cleansing, unique filtering, annotation, splitting, and analysis. The resulting dataset is in CoNLL format, annotated using the IOB2 scheme with six entity types. The total number of log messages acquired from 12 DJI drone models is 1850. The data are split based on the drone models, resulting in 1412 messages for training and 438 messages for testing. The average length of log messages is 6.5 globally, 6.6 and 8.8 for the train and the test sets, respectively.

**Specifications Table**

**Table:** 

Subject:	Cryptography and Cybersecurity
Specific subject area:	Named entity recognition, drone named entity recognition, information extraction for drone forensic investigation, NLP for digital forensics
Type of data:	Tabular (Extracted flight logs) Text (Extracted flight logs, annotated dataset)
How the data were acquired:	The primary data were downloaded from the VTO Labs under the Drone Forensic Program [1], which is publicly available in the form of drone forensic images. Most of the drone image data are encrypted. Therefore, Autopsy was used to decrypt the data. Python programming is used to build several scripts to parse the log message and timestamp information from the flight log data.
Data format:	Raw Flight Logs (.csv) Annotated in CoNLL format (.txt)
Description of data collection:	From the available drone images provided by VTO Labs, the DJI model is chosen as the source data after exploring the availability of flight log message information. All controller types are selected as the flight log data source, including Android-based, iOS-based, and tablet controllers. Flight log is the only log message source since the telemetry data contains no human-readable message [2]. All the parsed log messages are kept, and no duplicate removal is performed.
Data source location:	All the primary data were collected from VTO Labs under the Drone Forensic Program [1]
Data accessibility:	Repository name: Mendeley Data Data identification number: 10.17632/fwcjyc754h.1 Direct URL to data: https://data.mendeley.com/datasets/fwcjyc754h/1
Related research article:	S. Silalahi, T. Ahmad, H. Studiawan, Transformer-Based Named Entity Recognition on Drone Flight Logs to Support Forensic Investigation, IEEE Access. 11 (2023) 3257–3274. https://doi.org/10.1109/ACCESS.2023.3234605

## Value of the Data


 

• The constructed dataset is an attempt at the NER for flight logs drone forensic research. The dataset are expected to be useful for further research development on the employment of natural language processing-based methods to perform information extraction to assist drone forensic investigation. The entity types defined in this dataset can be an initial step in identifying more entity types related to drone incidents.
• Generally, the dataset construction would benefit the research community in the drone forensics domain, for the dataset can be used as a benchmark for NER model development. The raw data from the data collection and extraction [3] can also be utilized to conduct drone forensic research other than NER.
• The dataset is in CoNLL format, annotated using the IOB2 scheme as the default scheme. The further experiment can use another annotation scheme to test their proposed method and compare the performance. The tokenization used in this dataset still has room for improvement since several tokenization methods are available in the NLP research.
• There are two constructed datasets for the NER task annotated using contextual and consistent tagging procedures. Since the flight log contains critical information regarding a flight taken by a drone device, the raw flight logs that were successfully collected and decrypted can be used to perform another drone forensic research such as log mining [4], by utilizing the raw flight logs.

## 1 Objective

The research article written using this dataset is the first attempt to utilize NER in the drone forensic domain [5]. Publishing the dataset through this paper makes the research more verifiable and reproducible. The research article related to this dataset is intended to explore the feasibility of performing information extraction from the flight log data to assist a forensic investigation process [6]. As for this data article, the main purpose is to document the long process of drone flight log data collection and extraction to accelerate drone forensic research in utilizing flight logs as one of the primary forensic evidence [7].

## 2 Data Description

**Fig. 1:**
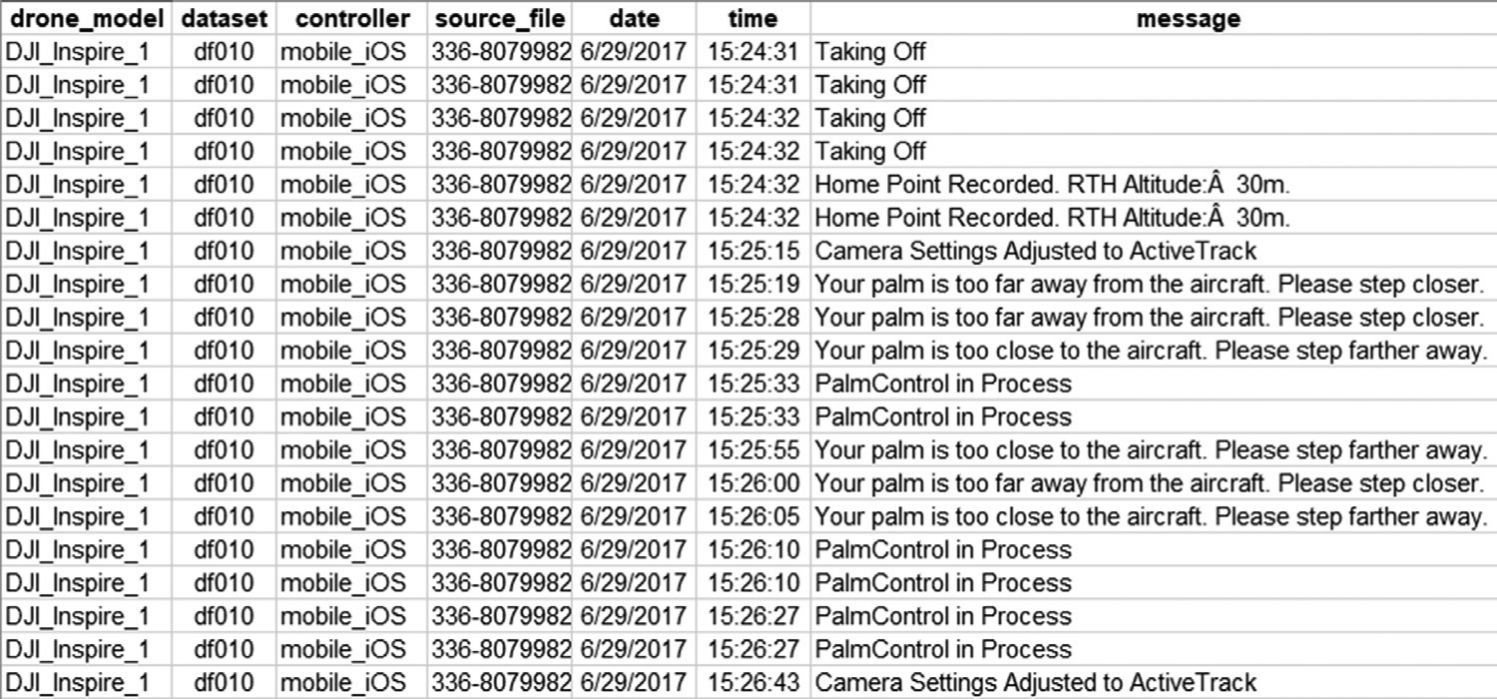
Forensic timeline constructed from flight logs (parsed.csv in raw folder).

During the flight, a drone device records lots of data regarding the device's condition, including the environmental situation. These data are called flight log data, where incident-related information is stored [7]. In a DJI-made drone, the flight log data commonly contain 169 columns. From those columns, only three of them contain a human-readable message describing the sequential events that happen to the drone during the flight, i.e., *message, tip,* and *warning.* These three columns are the main objective of data extraction to acquire the log message relevant to each event experienced by the drone device. The *warning* column informs the pilot about potential issues regarding the flight condition, such as hardware issues, environmental issues, and maximum constraints of some exceeded constraint parameters. As for the *tip* column, it contains some directive instructions of what could be taken by the pilot during the flight. Otherwise, the information is written in the *message* column. These three columns are collected into one single file ordered sequentially to form a forensic timeline, as shown in Fig. 1. Despite the other types of logs stored in another folder, none of them has human-readable as in the flight log message.

In order to acquire the information from the log file, as previously explained, the flight logs need to be extracted from the drone images first. Even if the same brand manufactures each drone type, it has its own location to store the flight log data. It depends on the controller device used to operate the drone as well. For instance, an Android-based controller device stored the flight log data in the /dji/dji.go.v4/FlightRecord or /dji/dji.pilot/FlightRecord directory. Flight log data in a DJI-made drone are stored in an encrypted .txt file that needs to be decrypted using their proprietary tools, named DJI Phantom Log Viewer^1^. Other than the paths mentioned above, the DJI flight log acquired from an Android-based controller can also be found either in the /dji/dji.go.v4/LOG/ERROR_POP_LOG directory or /dji/dji.pilot/LOG/ERROR_POP_LOG directory. Nevertheless, the stored log is not encrypted. After performing the data preparation, the resulting dataset has an average 6.6 and 8.8 words length for train and test sets. However, most messages range from one to ten in length, which is more than 80% of the total messages. The message length frequency distribution is shown in Fig. 5.

Unlike the Android-based controller, the iOS-based controller store the flight log in an encrypted file with no extension. The Autopsy is used to decrypt the flight log file originating from an iOS-based controller to get the plain version, then parse the human-readable message. Besides Android and iOS-based controller, tablet controller is also used to fly the drone. One of the primary data used a tablet controller to fly the DJI Matrice 210. The complete list of the primary data source used in this dataset is shown in Table 1.

**Table 1:** List of primary data used.

Drone type	Dataset	Year / Month	Evidence Source	File name
DJI Inspire 1	df010	2017 / 08	mobile_iOS	DF010_flight.zip
		2018 / 06	mobile_android	Media01.zip
			mobile_iOS	ios_backup.zip
	df011	2017 / 08	mobile_android	DF010_flight.001
			mobile_iOS	DF011_flight.zip
DJI Inspire 2	df025	2017 / 08	mobile_android_physical	df025_flight_android_physical.001
			mobile_iOS_backup	df025_flight_ios_backup.zip
	df026	2017 / 08	mobile_android_physical	df026_flight_android_physical.001
			mobile_iOS_backup	df026_flight_ios_backup.zip
	df027	2017 / 08	mobile_android_physical	df027_flight_android_physical.001
		2017 / 08	mobile_android_physical	df027_flight2_android_physical.001
			mobile_iOS_backup	df027_flight_ios_backup.zip
		2018 / 06	mobile_iOS_backup	ios_backup.zip
			mobile_android_logical	Android_logical.zip
DJI Matrice 210	df059	2018 / 06	controller_tablet_intact	Controller_tablet_intact_physical.001
	df060	2018 / 06	tablet_physical_intact	Tablet_Physical_Intact.001
DJI Matrice 600	df034	2018 / 06	mobile_android_logical	Android_logical.zip
			mobile_iOS_backup	ios_backup.zip
DJI Mavic 2	df067	2018 / 09	mobile_android_logical	Android_logical.zip
			mobile_iOS_backup	ios_backup.zip
	df068	2018 / 09	mobile_android_logical	Android_logical.zip
			mobile_iOS_backup	ios_backup.zip
	df069	2018 / 09	mobile_android_logical	Android_logical.zip
			mobile_iOS_backup	ios_backup.zip
DJI Mavic Air	df048	2018 / 04	mobile_android_logical	MC 04.zip
			mobile_iOS_backup	MC 04 iOS.zip
		2018 / 06	mobile_android_logical	Android_logical.zip
			mobile_iOS_backup	Mobile_iOS_Backup.zip
	df049	2018 / 04	mobile_android_logical	MC 05.zip
			mobile_iOS_backup	MC 05 iOS.zip
	df050	2018 / 04	mobile_android_logical	MC 06.zip
			mobile_iOS_backup	MC 06 iOS.zip
DJI Mavic Pro	df019	2017 / 08	mobile_android_physical	df019_flight_android_physical.001
			mobile_iOS_backup	df019_flight_ios_backup.zip
		2018 / 06	mobile_iOS_backup	ios_backup.zip
	df020	2017 / 08	mobile_android_physical	df020_flight_android_physical.001
			mobile_iOS_backup	df020_flight_ios_backup.zip
		2018 / 06	mobile_android_logical	Android_logical.zip
	df021	2017 / 08	mobile_android_physical	df021_flight_android_physical.001
			mobile_iOS_backup	df021_flight_ios_backup.zip
DJI Phantom 3	df001	2018 / 06	mobile_android	Media01.zip
			mobile_iOS	ios_backup.zip
	df002	2017 / 06	mobile_android_backup	DF002_flight.zip
			mobile_iOS_backup	DF002_flight.zip
	df003	2017 / 06	mobile_iOS_backup	DF003_flight.zip
DJI Phantom 4	df005	2017 / 06	mobile_android_backup	DF005_flight.zip
			mobile_iOS_backup	DF005_flight.zip
			mobile_iOS	ios_backup.zip
	df006	2017 / 06	mobile_android_backup	DF006_flight.zip
			mobile_iOS_backup	DF006_flight.zip
DJI Phantom 4 Pro V2	df061	2018 / 06	mobile_android_logical	mobile_android_logical.zip
			mobile_iOS_backup	mobile_ios_backup.zip
	df062	2018 / 06	mobile_android_logical	DF062 305.zip
			mobile_iOS_backup	Phantom4ProV2DF062.zip
	df063	2018 / 06	mobile_android_logical	Media01.zip
			mobile_iOS_backup	mobile_ios_backup.zip
DJI S1000+	df043	2018 / 01	mobile_android_logical	Mobile_Android_Logical.zip
			mobile_iOS_backup	ios_backup.zip
DJI Spark	df007	2018 / 06	mobile_iOS	ios_backup.zip
	df008	2017 / 06	mobile_iOS_backup	DF008_flight.zip
		2018 / 06	mobile_iOS	ios_backup.zip

**Fig. 2:**
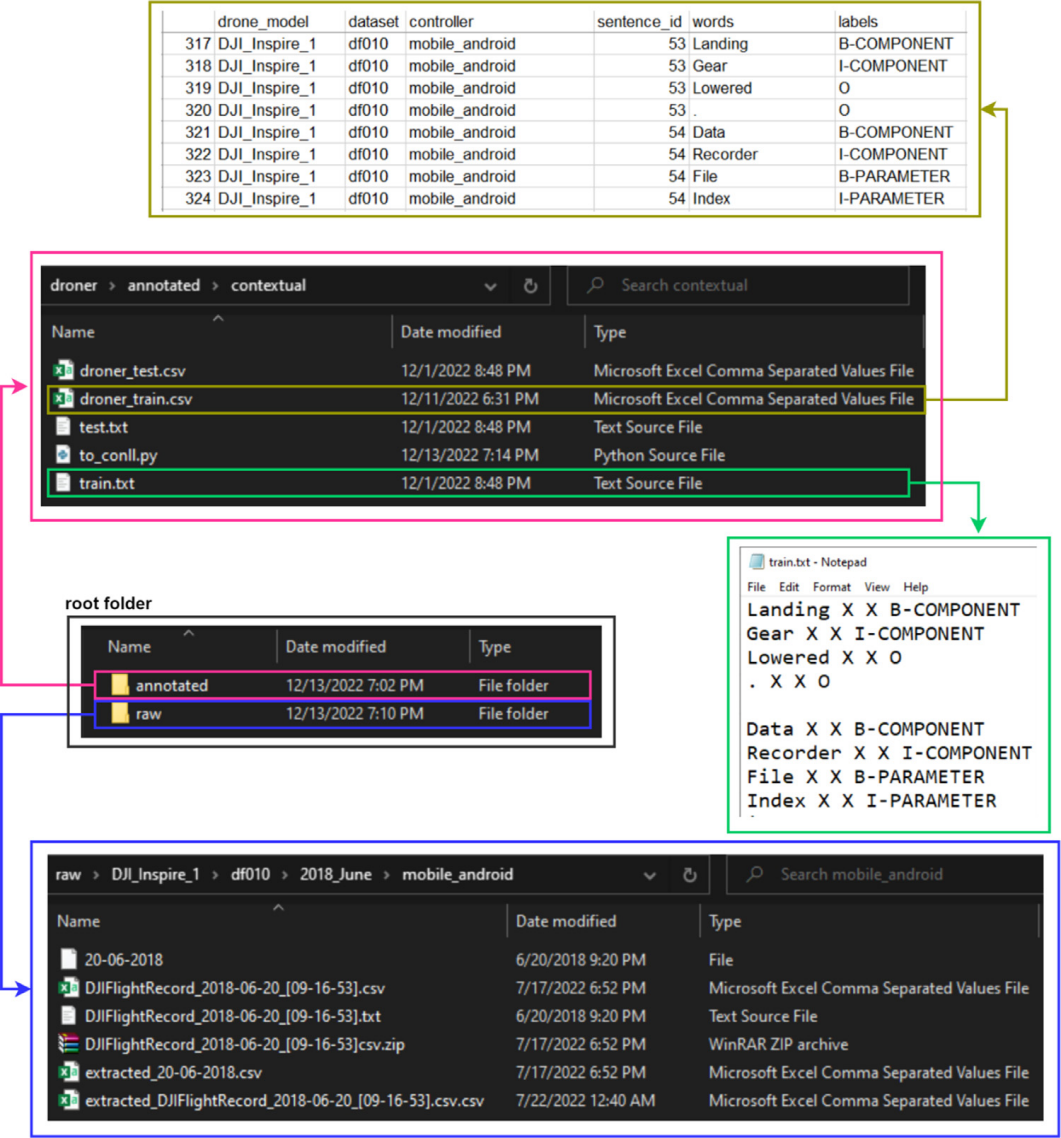
The collected raw flight logs and NER annotated datasets.

**Fig. 3:**
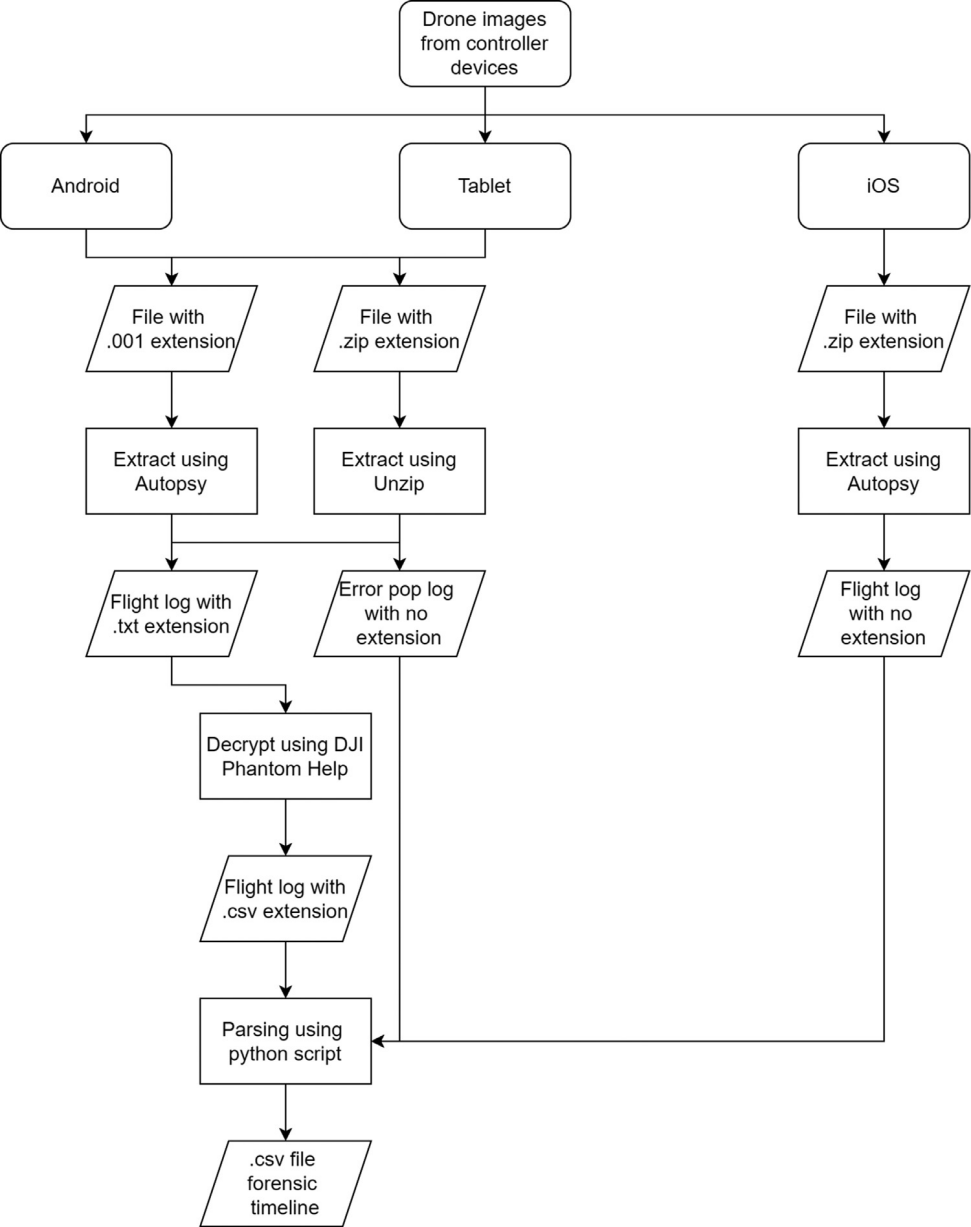
Data extraction pipeline.

In sequence labelling tasks, CoNLL is one of the most commonly used formats for the dataset. It consists of tokens and labels consecutively in a txt file. Fig. 2 shows a sequence in CoNLL format that is annotated using consistent and contextual tagging procedure in the IOB2 annotation scheme. The annotation procedure is explained in the following section. The train.txt and test.txt files are formatted in 4 columns, as shown in Fig. 3, using a python script (*to_conll.py*) to follow the CoNLL2003 dataset for code compatibility reasons. The metadata of every flight log is preserved to open a space for analysis, such as different splitting, grouping, or filtering criteria of the collected data. The previously mentioned metadata refers to the drone model, drone image identifier, and the controller name from which the log messages originated. Fig. 3 shows the structure of the dataset folder. The annotated NER datasets using the contextual and consistent tagging procedures are stored in the annotated folder. The raw folder contains the encrypted, decrypted, and extracted flight logs. The decrypted file contains the complete flight information, while the extracted file contains the timestamp and the message with additional fields, such as drone model, dataset identifier, controller device type, and flight log file name. Since the decrypted raw flight logs contain lots of flight information, including sensor data, the collected raw dataset can be used by the research community to conduct research other than NER in the drone forensics domain.

## 3 Experimental Design, Materials and Methods

The absence of the NER dataset in the drone forensic domain is one of the motivations of this paper, to make the dataset publicly available to the research community. Unlike the other NER dataset gathered from wiki articles or public news, the NER dataset constructed in this paper was gathered from a number of drone flight logs extracted from several drone forensic images provided by the VTO Labs Drone Forensic Program [8]. This chapter explains the process of constructing the dataset from data collection to annotation, along with the corresponding code used to perform each stage/phase. The flow of the extraction process is shown in Fig. 3.

Before exploring all the drone images listed in the previous section, we first explore two drone images taken from the DJI Inspire 1 model, i.e., DF010 and DF011. After exploring all the possible paths of drone flight log location and documenting the steps taken, those steps are applied to all drone images, further explained in the following sub-sections.

The first step was data collection. In this step, we collect all the drone forensic images acquired from the controller devices available in every DJI Model. Three different controllers were discovered, i.e., Android-based, iOS-based, and tablet controllers. Drone forensic images acquired from an Android-based controller are stored in .001 or .zip files. For a file with a .001 extension, we use Autopsy to mount the disk and explore the folder structure to find the flight log files. The file with a .zip extension can be directly extracted. After we found the flight log with a .txt extension, we used DJI Phantom Help tools to decrypt the file and yielding a .csv file. The decrypted file contains lots of flight information, including log messages which then being parsed using a python script (*parse_android.py*) we have developed. Another flight log stored in the LOG folder and not encrypted is parsed to get the log message along with the timestamp to build the forensic timeline.

The drone forensic images acquired from iOS-based controllers have a completely different folder and file structure from the Android-based controller. The flight log is stored in files with no extension inside several folders with a flat structure. We use Autopsy to decode the file contents and find that some files contain flight log messages. We use a python script (*parse_ios.py*) to parse the log message and construct the drone forensic timeline out of it. The last type of controller is the tablet controller, which is stored in files with a .001 extension. We found the same behaviour as the images acquired from the Android-based controller. After parsing all the found flight logs from all DJI models, we collect all the log messages along with the corresponding timestamps into a single .csv file using a python script (*parse_csv.py*) we developed. All the scripts mentioned in this paper are hosted in the data repository.

The next step is cleansing the log messages. Some messages contain unknown characters as a result of the decryption process. We carefully read all the messages and removed every found unknown character. We also preprocessed several log messages having an inconsistent format to avoid inconsistency in the next steps. Several inconsistencies that are found include the use of different spacing after a colon. Some messages use a space after a colon, yet others do not use a space after a colon. This is found in the “Return-to-Home Altitude: 65FT” message. Some messages start with open quotation marks and need to be cleansed so that all the messages are in the same format. Another case of cleansing is missing the period between two sentences in a single log record. So we need to add a period to separate the two sentences.

After cleansing the collected log messages, the next step is identifying the entity type that exists in drone flight log data. The entity type is the class name in the NER problem that is used to label the region of interest in text data that is usually given to each word. After comprehensively reading all the log messages, we identify six entity types, i.e., Action, Component, Function, Issue, Parameter, and State. These six entity types are used as the tagset in the annotation process. Before performing the annotation process, we first define several rules to be used as a reference or guidance in giving a label to each word so that the final annotated dataset has a consistent label in terms of context.

Here are the rules used to perform the data annotation process. An action label is given to a word or phrase indicating an action the drone took during a flight, i.e., return to home, taking off, and landing. The second entity type is component. The component label is given to span that indicates an aircraft's physical component. Several samples of this entity type are landing gear, motor, and battery. Function is an entity type representing features or functionalities available in a certain type of drone. Obstacle avoidance, obstacle sensing, and intelligent flight are a sample of spans that belong to the function entity type. In case the drone is experiencing some issues, the log records will be generated. For this type of message, the label issue is given. Compass interference, command timeout, and downlink lost are some samples of a span that belong to the issue entity type. The next entity type is the parameter. Parameter is an entity type representing some configuration setting in the drone to be used in a flight, i.e., max flight distance, max altitude, home point, and minimum RTH altitude. The last entity type is the state entity type. State is an entity type indicating a drone's mode while flying. GPS mode, atti mode, sport mode, and auto landing mode are a sample of span given the state label.

After the annotation rules are defined, the next step is data annotation. We use the tecoholic^2^ tools to tokenize and annotate the data. We preserve the log message as written originally without lowercasing them to allow the model to see the difference between words written in capital case and lowercase. The punctuation (dot and comma) are also preserved, as they play the context separator role in a sentence. After tokenizing the messages, we manually annotate the unique message in the dataset. Several annotation schemes are commonly used in the NER dataset, and one of the most widely used is IOB2 [9]. IOB scheme has three different tags, i.e., inside, outside, and beginning. The beginning tag is given to the first word of mentioned entity, and the consecutive words of the same entity type are given the inside tag. For example, “battery temperature” is given B-Parameter and I-Parameter, respectively. For non-entity words are given the outside tag. The sample of the final annotated dataset in CoNLL format is shown in Fig. 3. The resulting annotated unique message is then used as a lookup dictionary to annotate the rest of the messages.

**Fig. 4:**
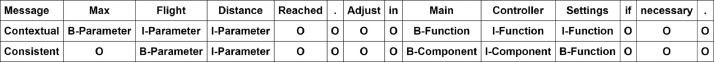
The comparison of annotation results from contextual and consistent tagging procedures.

Despite the annotation rules that have been defined, some ambiguous spans in the dataset can alternatively belong to several different entity types. Therefore, we investigate two different tagging approaches to annotate the dataset, namely consistent and contextual tagging procedures. For contextual tagging, we choose the longest span as the context to determine the entity type and the label given to the span. For instance, “battery temperature” is given the parameter label for both words instead of the component label for the “battery” and the outside label for the “temperature”. By means, the surrounding words determine the context of a word along with the entity type. Therefore, a particular word likely belongs to a different label for each distinct context within different sentences. Contrarily, for consistent tagging, the shortest span is used as the context to determine the entity type that a particular word belongs to. By means, it is unlikely that a word is assigned different labels within different sentences. If a single word has been assigned a particular label, the surrounding words will not change the context for that word, leading to a change of entity type to which the word belongs to. For example, the word “battery” belongs to the Component entity type regardless of the surrounding words. Fig. 4 shows the contrast between the results of the two annotation approaches.

**Fig. 5:**
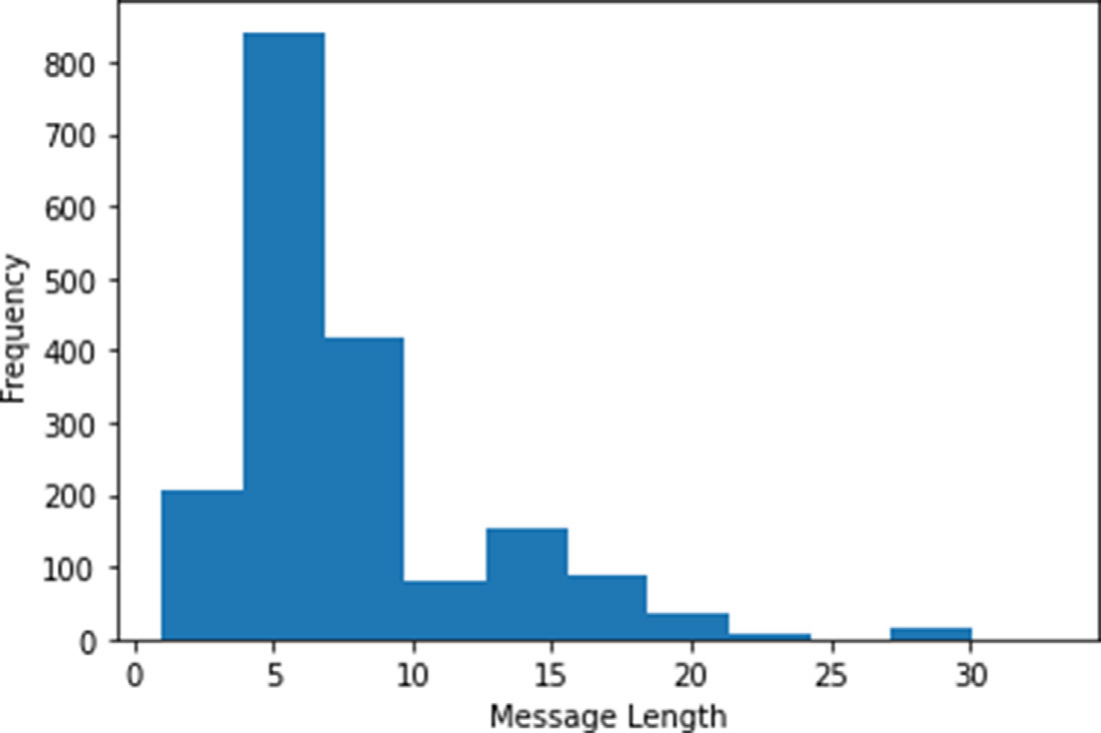
Message length frequency distribution in the dataset.

After completing the annotation process for all the log messages, the dataset was split into train and test sets. Usually, the splitting technique uses random sampling with a certain proportion percentage. However, for our dataset, we split the dataset based on the drone model. As shown in Table 4, the first nine drone models are used as the train set, while the last four drone models are used as the test set. The reason behind this splitting technique is to separate the unique log message based on the drone model, as each drone model has its own features which differ from one to another. Assuming that more advanced drone models have more advanced features, which then generate different log messages compared to less advanced ones, the test set contains messages that do not exist in the train set. As the annotation process has been completed, the annotation result is formatted to the CoNLL structure using a python script (*to_conll.py*). The final composition of the dataset for each train and test set resulted from two annotation procedures is shown in Table 2. The distribution of each entity type is displayed in Table 3. The final proportion between the train and the test is 76:24.

**Table 2:** The number of messages, tokens, and entities in the dataset.

Dataset	Split	Message	Token	Entities
Consistent	Train	1412	9103	3843
	Test	438	4005	953
	Total	1850	13108	4796
Contextual	Train	1412	9103	4184
	Test	438	4005	1118
	Total	1850	13108	5302

**Table 3:** The distribution of entities per entity type.

Dataset	Split	Component	Parameter	Function	State	Issue	Action
Consistent	Train	566	1042	830	134	791	480
	Test	175	206	123	124	125	200
	Total	741	1248	953	258	916	680
Contextual	Train	503	1828	490	125	955	283
	Test	147	322	167	107	246	129
	Total	650	2150	657	232	1201	412

**Table 4:** The number of messages acquired from every drone model.

Drone Model	Dataset	# of message
DJI Mavic 2 Pro	df069	28
DJI Mavic 2 Zoom	df068	117
	df067	31
DJI Matrice 210	df060	49
	df059	34
DJI Mavic Air	df050	85
	df048	101
DJI_S1000+	df043	17
DJI_Matrice_600	df034	96
DJI_Inspire_2	df027	249
	df026	109
	df025	83
DJI_Mavic_Pro	df021	54
	df020	161
	df019	92
DJI_Inspire_1	df011	44
	df010	62
DJI_Phantom_4_Pro_V2	df063	64
	df062	63
	df061	46
DJI_Spark	df008	31
	df007	3
DJI_Phantom_3	df002	34
	df001	59
DJI_Phantom_4	df006	62
	df005	76
Total		1850

## Ethics Statements

This work does not involve things like human subjects, animal experiments and data collection from social media platforms.

## CRediT authorship contribution statement

**Swardiantara Silalahi:** Conceptualization, Data curation, Formal analysis, Investigation, Methodology, Software, Validation, Visualization, Writing – original draft. **Tohari Ahmad:** Conceptualization, Funding acquisition, Methodology, Project administration, Supervision, Writing – review & editing. **Hudan Studiawan:** Conceptualization, Formal analysis, Investigation, Methodology, Resources, Supervision, Validation, Writing – review & editing.

## Declaration of Competing Interest

The authors declare that they have no known competing financial interests or personal relationships that could have appeared to influence the work reported in this paper.

## Data Availability

DroNER: Dataset for Drone Named Entity Recognition (Reference data) (Mendeley Data).

## Footnotes

1 https://www.phantomhelp.com/logviewer/upload/

2 https://tecoholic.github.io/ner-annotator/
